# Sex differences in cued fear responses and parvalbumin cell density in the hippocampus following repetitive concussive brain injuries in C57BL/6J mice

**DOI:** 10.1371/journal.pone.0222153

**Published:** 2019-09-05

**Authors:** Laura B. Tucker, Brian S. Winston, Jiong Liu, Alexander G. Velosky, Amanda H. Fu, Antigone A. Grillakis, Joseph T. McCabe

**Affiliations:** 1 Pre-Clinical Studies Core, Center for Neuroscience and Regenerative Medicine, Uniformed Services University of the Health Sciences, Bethesda, Maryland, United States of America; 2 Department of Anatomy, Physiology & Genetics, F.E. Hébert School of Medicine, Uniformed Services University of the Health Sciences, Bethesda, Maryland, United States of America; 3 Graduate Program in Neuroscience, F.E. Hébert School of Medicine, Uniformed Services University of the Health Sciences, Bethesda, Maryland, United States of America; Radboud University Medical Centre, NETHERLANDS

## Abstract

There is strong evidence to suggest a link between repeated head trauma and cognitive and emotional disorders, and Repetitive concussive brain injuries (rCBI) may also be a risk factor for depression and anxiety disorders. Animal models of brain injury afford the opportunity for controlled study of the effects of injury on functional outcomes. In this study, male and cycling female C57BL/6J mice sustained rCBI (3x) at 24-hr intervals and were tested in a context and cued fear conditioning paradigm, open field (OF), elevated zero maze and tail suspension test. All mice with rCBI showed less freezing behavior than sham control mice during the fear conditioning context test. Injured male, but not female mice also froze less in response to the auditory cue (tone). Injured mice were hyperactive in an OF environment and spent more time in the open quadrants of the elevated zero maze, suggesting decreased anxiety, but there were no differences between injured mice and sham-controls in depressive-like activity on the tail suspension test. Pathologically, injured mice showed increased astrogliosis in the injured cortex and white matter tracts (optic tracts and corpus callosum). There were no changes in the number of parvalbumin-positive interneurons in the cortex or amygdala, but injured male mice had fewer parvalbumin-positive neurons in the hippocampus. Parvalbumin-reactive interneurons of the hippocampus have been previously demonstrated to be involved in hippocampal-cortical interactions required for memory consolidation, and it is possible memory changes in the fear-conditioning paradigm following rCBI are the result of more subtle imbalances in excitation and inhibition both within the amygdala and hippocampus, and between more widespread brain regions that are injured following a diffuse brain injury.

## Introduction

In recent years research efforts have increased toward understanding the relationship between repetitive concussive brain injuries (rCBI) and delayed neurodegenerative brain conditions characterized by symptoms including emotional dysregulation (i.e., depression, anxiety, irritability) and cognitive dysfunction [1]. The amygdala and hippocampus are brain regions linked to depression and stress/anxiety-related disorders in non-brain-injured populations (e.g., [2–4]), and it has been long-accepted that the hippocampus is a critical brain region for information processing related to learning and memory. Accordingly, pathologies in these brain regions have been described in patients who have sustained repetitive brain injuries and suffer neuropsychiatric and cognitive symptoms such as depression and memory loss [5].

Animal models of rCBI have been developed that enable the study of functional deficits that evolve following multiple injuries [6–10], and pre-clinical traumatic brain injury (TBI) models also afford the opportunity to probe the neural mechanisms underlying behavioral dysfunction following injuries [11–15]. The fear conditioning (FC) behavioral paradigm, in which a rodent learns to associate a neutral context and auditory tone (conditioned stimuli; CS) with an aversive stimulus (unconditioned stimulus; US), has been employed in translational studies to model functional deficits following injury to study aspects of hippocampal- as well as amygdala-dependent memory [11, 15, 16]. The neural circuits underlying defensive responses (typically measured by freezing behavior in response to a CS) have been well-defined (e.g., [17]) and as such, *contextual FC*, where the animal is placed back into the context in which it learned to associate the CS with the US (in the absence of the US), is typically employed to test hippocampal-dependent cognitive function, whereas *cued FC*, in which the cue (CS) is presented in a neutral context, is presumed to test amygdala function.

There is clinical evidence that despite progesterone and estrogen conferring a neuroprotective effect [18–20], women may be more likely to develop a neuropsychiatric disorder following a TBI [21–23]. Despite increasing efforts in recent years to be more inclusive of both sexes in pre-clinical TBI behavioral research (e.g.,[7, 24–29]), there is still a need to better understand behavioral consequences, particularly neuropsychiatric symptoms, in both sexes following TBI and rCBI. The goal of the current study was to employ the cued and context FC paradigm to assess amygdala- and hippocampal-related fear learning in both male and female mice, and also to assess anxiety- and depressive-like behaviors in the elevated zero maze and tail suspension test, respectively, following experimental rCBI.

## Materials and methods

### Animals

Male and female C57BL/6J mice 8 weeks old were obtained from The Jackson Laboratory (Bar Harbor, ME) and allowed to acclimate to Association for Assessment and Accreditation of Laboratory Animal Care-accredited housing facilities for approximately one week before TBI procedures. At the time of the first injury or sham procedure, male and female mice weighed an average of 26.91 g and 19.40 g, respectively. Mice were group-housed in same-sex cages (3–5 per cage); rodent chow (Harlan Teklad Global Diets 2018, 18% protein) and water were available *ad libitum* and the room was on a standard 12-h light-dark cycle. Fear conditioning testing was performed by a male investigator, all other behavioral testing and TBI procedures were carried out by female investigators [30]. All described procedures were approved by the institutional animal care and use committee at the Uniformed Services University of the Health Sciences (Bethesda, MD).

### Repetitive concussive brain injury (rCBI)

Repetitive concussive brain injury (rCBI) procedures were performed as previously described [7, 11]. Mice were randomly assigned to receive rCBI (3x) or sham (3x) procedures at 24-hour intervals and were further divided into shocked and non-shocked controls when fear conditioning procedures began (Table 1). Mice were anesthetized in a clear induction chamber with 3% isoflurane (Forane, Baxter Healthcare Corporation, Deerfield, IL) in 100% oxygen until corneal and pedal reflexes were absent. Head hair was clipped and Nair hair-removal cream (Church & Dwight, Princeton, NJ) applied to remove all fur from the scalp. The mouse was then positioned in a stereotax with atraumatic ear bars and an incisor bar where anesthesia (1.5% isoflurane) was maintained via a flow-through nose cone. The suture of the cranium was located under bright illumination and a permanent marker was used to mark the location with a small dot. The steel tip of the impactor (5.0-mm) was centered over the injury site (2.5 mm left of bregma, 2.5 mm posterior to bregma) at a 15° angle relative to the sagittal plane. A Leica Microsystems (Buffalo Grove, IL) Impact One^™^ device was employed to deliver concussive impacts. Auditory feedback from the impact device confirmed primary contact with the skin. Anesthesia was discontinued immediately prior to impact (with continuation of oxygen); impact was delivered with velocity 5.0 m/s, dwell time 0.1 s and depth of 1.2 mm. Any occurrence of apnea following the injury was measured; mice were then placed into a clean cage on a warming pad in a supine position. The amount of time required to return to a prone position was recorded as the righting reflex. Sham-treated mice underwent all procedures with the same durations of anesthesia (approximately 10 min on day 1, 6 min on days 2–3), except the impact.

**Table 1:** Number of mice in each group.

	Male		Female	
	Shock	No Shock	Shock	No Shock
rCBI	17	12	18	12
Sham	18	12	18	12

rCBI, repetitive concussive brain injury

### Behavioral studies

**Fig 1:**
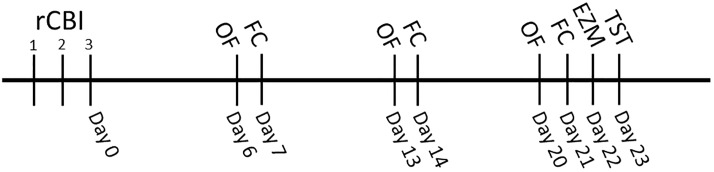
Experimental timeline. Concussive brain injuries, repeat (rCBI), were performed on three consecutive days, with mice sustaining one injury each day. Mice were tested in the open field (OF) arena on days 6, 13 and 20 following the final CBI. Fear conditioning (FC) association (footshock/auditory stimulus pairings) took place one week following rCBI, with context and cue testing one and two weeks later. Testing for anxiety- and depressive-like behaviors were performed in the elevated zero maze (EZM) and tail suspension test (TST), respectively, on days 22 and 23 following injuries.

The timeline of behavioral testing with respect to rCBI is outlined in Fig 1. Mice were tested in the open field (OF) on days 6, 13 and 20 following the final CBI or sham procedure (day 0). Fear conditioning (FC) association (pairing of neutral tone with aversive foot shock) took place on day 7 following injury; context and cued tests were performed on days 14 and 21. Anxiety- and depressive-like behaviors were tested on days 22 and 23 in the elevated zero maze (EZM) and tail suspension test (TST), respectively. Order of tests was generally intended to be least stressful to most stressful, with the TST being the final test. Bodden and colleagues recently demonstrated that repeated OF testing has no impact on multiple behavioral and physical measures of stress in mice [31], and test order has been shown to have no effect on FC and OF testing in a large battery of behavioral testing [32]. However, as FC requires administration of shock and was performed relatively early, unshocked controls were employed in the experimental design to determine the effects of the early shocks on subsequent behavioral results.

The experiment was performed in multiple cohorts balanced by both sex and injury. Within each cohort, females were tested prior to males in the OF, EZM and TST. During FC, unshocked mice were tested first to avoid effects of stress pheromones. This, in addition to having multiple trials on days 14 and 21, required groups of females and males to be tested in alternation. All behavioral equipment was thoroughly cleaned with 70% ethanol and allowed to dry between trials of individual mice.

#### Open field (OF)

Open field (OF) testing was performed as previously described [10] in 40 cm x 40 cm OF arenas with a light level of approximately 5 lux and opaque walls 35 cm high (Stoelting Co., Wood Dale, IL). Each OF arena (8 available) was equipped with an overhead camera and connected to a computer with Any-Maze software (Stoelting) that tracked the mouse during a 20-min session. Mice were placed in the center of the apparatus and the software recorded the total distance traveled and the distance traveled in a software-defined center zone (20 cm x 20 cm) of the apparatus, expressed as a percentage of the total distance traveled.

#### Fear conditioning (FC)

The methods described by Logue and colleagues [11] following rCBI were employed in the current study. On day 7 following the final rCBI or sham procedure, mice were trained to associate a neutral/conditioned stimulus (the context and the cue/tone) with an aversive/unconditioned stimulus (foot shock). Mice were placed into Plexiglas chambers (17 cm x 17 cm, 4 lux; Ugo Basile, Varise, Italy) with salient black and white checkerboard or striped walls, and mint or citrus odor cues. Mice acclimated to the chambers for two minutes, after which a 30-s tone (3 KHz, 80 dB) co-terminated with a 2-s, 0.5 mA foot shock. After an interval of 1 min, the tone and foot shock pairing was delivered a second time. After a final 1-min monitoring period, the test ended. Mice were placed into holding cages until all mice in a cage were tested, after which they were all returned to the home cage.

Seven and 14 days later (14 and 21 days following injury or sham procedures), context- and cue-dependent memories were tested. First, context-dependent memory was assessed by returning the mice to the identical chamber in which they had previously associated the context with the foot shock; freezing behavior was assessed over a 5-min session. No less than one hour later, the context was altered with modified visual, light, tactile and odor (mint or citrus, order counter-balanced between mice) cues and mice were returned to the chambers for cue testing. After an acclimation period of 3 min, the tone from the training session was presented for 3 min, after which the animals were monitored for a final 1-min. During all fear conditioning testing, cameras in each chamber monitored mouse movements and Any-Maze software was employed for automated freezing detection (Any-Maze default settings: minimum freeze duration– 250 ms; freezing on threshold– 30; freezing off threshold– 40).

#### Elevated zero maze (EZM)

Mice were tested in the EZM on post-injury day 22 as previously described [24, 33]. The EZM (Stoelting) is an annular platform (60 cm in diameter) elevated 49 cm from the floor and divided into four equal quadrants. Two opposing quadrants were enclosed by opaque walls 16 cm high (“closed” quadrants) and the remaining two opposite quadrants were open with no walls but surrounded by a low edge approximately 1 cm high (“open” quadrants). Additional lighting was provided by overhead fluorescent lamps; the light levels in the closed and open quadrants were approximately 200 lux and 1600 lux, respectively. To begin the test session (5 min), mice were individually placed at the boundary of an open and closed quadrant, facing the inside of the closed quadrant, and allowed to freely explore the maze. An overhead camera linked to a computer with Any-Maze software tracked the movement of the mouse for the duration of the test.

#### Tail suspension test (TST)

The TST was performed on post-injury day 23 as previously described [10, 34]. Mice were suspended from laboratory shelves by their tails (approximately 1 cm from the tip of the tail) with standard laboratory tape approximately 25 cm long and 1.27 cm wide. Partitions were placed between individual mice to prevent visual interference and padding was placed on the surface below in the event of a fall. To prevent tail climbing, cylindrical polycarbonate tubing 4 cm in length, 1.3 cm inside diameter, 1.6 cm outside diameter (McMaster-Carr, Santa Fe Springs, CA; #8585K41) was placed around the tails. Test sessions were 6 min in duration and recorded with a standard video camera. Video files were later imported into Any-Maze software and the amount of time each mouse spent completely immobile was scored with a key press by an investigator blinded to the injury and shock status of the animals.

### Pathological assessment of injury

On day 28 following injury, all mice were deeply anesthetized and transcardially perfused with 0.1M phosphate buffer followed by 4% paraformaldehyde (PFA) in 0.1 M phosphate buffer. Brains were post-fixed for approximately 24 hr in 4% PFA, then transferred to 20% sucrose for 48 hr for cryoprotection. Brains were frozen and sectioned with a sliding microtome; sections (30 μm) were stored in a cryoprotectant solution at -20°C until processing.

#### Immunohistochemistry

Six mice from each injury and sex group were randomly selected for GFAP or parvalbumin immunohistochemical analysis. GFAP and parvalbumin immunohistochemistry was performed on free-floating sections as previously described [7] using mouse monoclonal antibodies (GFAP: 1:500, Thermo Fisher Scientific, MS-280-P; Parvalbumin: 1:10,000, Swant^®^ (Switzerland), 235). Free-floating sections were washed three times (10 min each) with 1x TBS-Triton (0.05%) and endogenous peroxidases were inactivated by H_2_O_2_ (0.3%) incubation for 30 min at room temperature (RT). The H_2_O_2_ was removed and the sections were washed again, then blocked in blocking buffer (10% goat serum + 2% bovine serum albumin in TBS-Triton (0.2%)) for 1 hr (RT). The primary GFAP or parvalbumin antibody (diluted in blocking buffer) was added to the sections and incubated overnight at 4°C. The following day, the antibody was removed, the sections were washed and the biotinylated secondary antibody (1:500 (diluted in blocking buffer); AffiniPure Goat Anti-Mouse IgG [H + L], Jackson ImmunoResearch Laboratories, 115-065-003) was added to the sections and incubated (RT) for 1 hr. The secondary antibody was removed, and the sections washed, then incubated in ABC reagent (Vector Labs, PK-4000) for 45 min (RT) and washed again. DAB staining solution (Vector Labs, SK-4100) was added and incubated for 3 min (RT), until sections were sufficiently visualized. Phosphate-buffered saline was added to stop the DAB reaction. Coronal sections from approximately Bregma to -1.80 mm [35] were mounted on slides and allowed to dry overnight, after which they were dehydrated through graded ethanol washes, cleared in xylene, and coverslipped with Permount.

**Fig 2:**
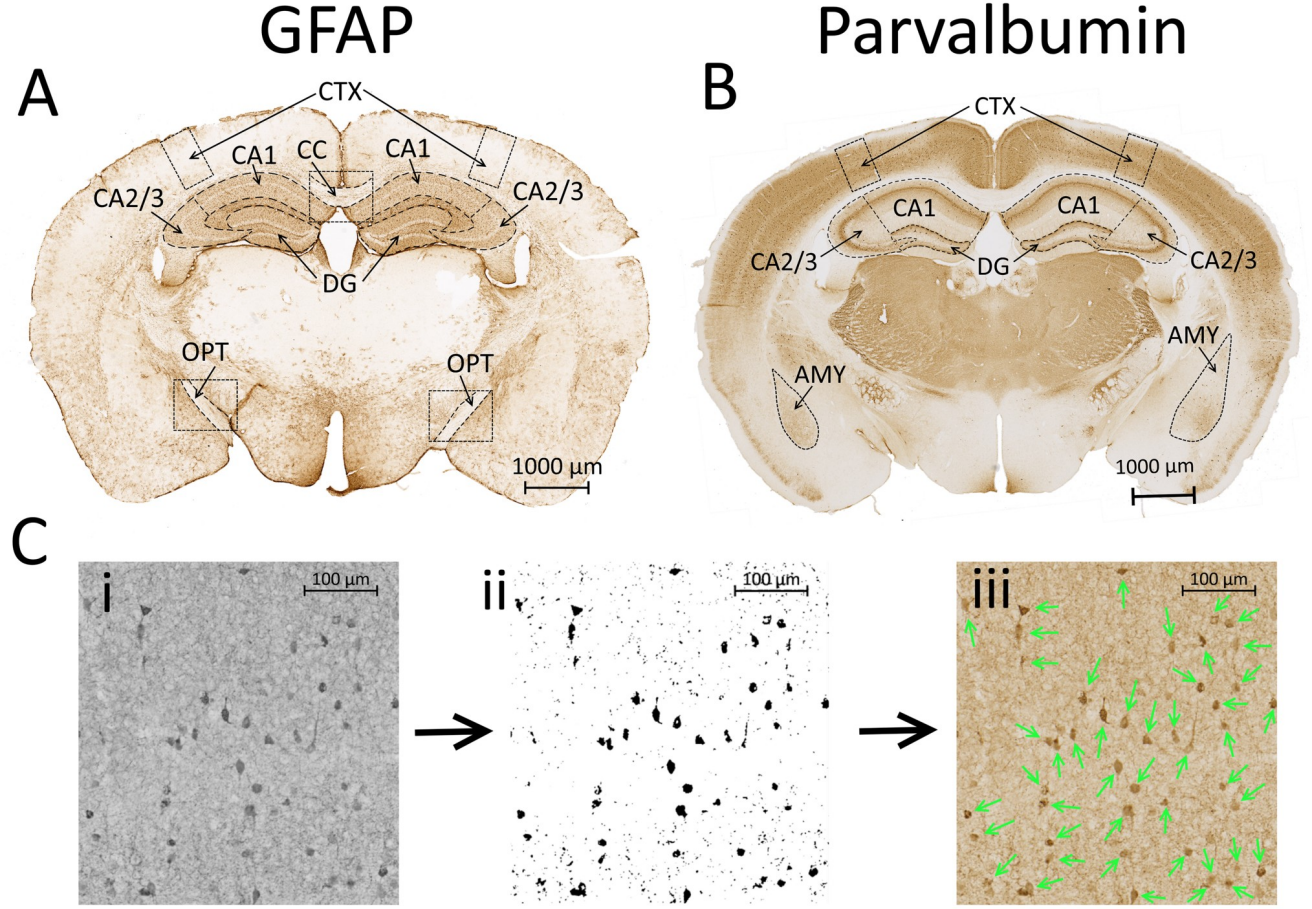
Glial fibrillary acidic protein (GFAP; **A**) staining density was analyzed in the corpus callosum (CC) and bilaterally in the perilesional cortex (CTX), hippocampus (HP; subdivided into CA1, CA2/3 and dentate gyrus (DG) regions) and optic tracts (OPT). Parvalbumin cell density (**B**) was quantified bilaterally in the amygdala (AMY), perilesional CTX and DG, CA1 and CA2/3 subregions of the HP. Fig. **2C** illustrates, in a sample of CTX, the process employed for counting parvalbumin cells. Panel **ii** shows the thresholded image from the black and white sample (**i**); the counted cells using the particle analysis feature of ImageJ are marked with arrows on the original image in (**iii**).

Slides were scanned with a Zeiss AxioScan Z1; images of areas of interest were captured and imported into ImageJ. GFAP density was quantified in the corpus callosum and bilateral hippocampus (divided into CA1, CA2/3 and dentate gyrus (DG)), optic tracts and perilesional cortex (500 μm wide region extending through the depth of cortex, 2500 μm from midline) as described previously [7] (Fig 2A). The mean grey density was determined with the measurement feature of ImageJ; the background (density of an area with no immunostaining) was subtracted. The density for each animal per region was typically averaged across three sections.

Parvalbumin cells were counted in bilateral amygdala, perilesional cortex (500 μm wide region extending through the depth of cortex, 2500 μm from midline) and the hippocampus (divided into CA1, CA2/3 and DG) (Fig 2B). The auditory cortex in an additional set of parvalbumin-stained sections ranging from Bregma -2.5 mm to -3.4 mm was also processed for parvalbumin cell density. The particle analysis feature of ImageJ was employed to count cells on 8-bit, thresholded images (minimum size: 150 pixel units, circularity 0.0–1.0) similar to methods previously described [36]. Most brain regions were thresholded using the Yen method (Fig 2C); a fixed lower threshold of 0.5% was employed for the CA2 region due to tissue heterogeneity of this region. Parvalbumin cell density for each region was typically averaged over three sections per animal. All GFAP and parvalbumin analyses were performed by an investigator blinded to the experimental conditions of the animals from which the sections were taken.

### Statistical analyses

Statistical analyses were performed with SPSS (version 20; IBM SPSS Statistics, Armonk, NY) and SAS Studio 3.6 (SAS Institute Inc., Cary, NC) software. Apnea and righting reflex data did not pass the homogeneity of variance test as assessed by Levene’s Test of equality of error variance; non-parametric Kruskal-Wallis tests were performed for each individual injury day followed by Dunn-Bonferroni-corrected post-hoc multiple comparisons (SPSS).

Parvalbumin and GFAP quantitative data were analyzed separately in mixed models (PROC mixed, SAS) with injury and sex as fixed factors and side as a repeated measure where appropriate. GFAP analysis and parvalbumin cell density counts in the hippocampus included region (DG, CA1, CA2/3) as a repeated measure. Interaction effects were followed up with Bonferroni-corrected planned contrasts (*t*-test).

FC training/association (day 7 post-injury), context and cue (days 14 and 21 post-injury) data were analyzed separately. Training data were transformed to cube root values to minimize violations of homogeneity of variance, and analyzed in a mixed model (PROC mixed, SAS) with injury, sex and shock status as fixed variables and time segment (10, 30-s segments) as a repeated measure. Context data were also transformed to cube root values and analyzed in a mixed model (SAS) with injury, sex and shock status as fixed variables, and day (post-injury day 14 and 21) as a repeated measure. Cue test data were transformed to square root values and analyzed separately for each day of the test (days 14 and 21 post-injury). Mixed models were employed with injury, sex and shock as fixed variables, and minute of the test as a repeated measure.

Where significant minute or day by shock interaction effects were found, post-hoc planned comparisons were performed to compare shocked vs. unshocked groups at each day or minute; *p*-values reported from these *t*-tests are Bonferroni-adjusted. Also, where significant injury by sex interactions were found, planned comparisons detected differences between injured and sham-treated mice of the same sex; these reported *p*-values are also Bonferroni-adjusted.

OF data (total distance traveled (transformed to natural log values) and activity in the center) were also analyzed in a mixed model (PROC mixed, SAS); injury, sex and shock status were fixed factors with post-injury day (6, 13, 20) as a repeated measure. Bonferroni-adjustments have been made to reported *p*-values resulting from comparisons between performance on individual days of OF testing. For all mixed models the Kenward-Roger degrees of freedom approximation was employed. Compound symmetry covariance structures provided the best fit for OF and FC context data; autoregressive Lag-1 covariance structures were employed for FC training and cue test data.

Data from tests performed at one time point (EZM and TST) were analyzed with a three-way analysis of variance (ANOVA; PROC GLM, SAS) with injury, sex and shock status as fixed factors. Finally, following statistically significant main effects or planned contrasts, effect size (Cohen’s *d*) was calculated as $\left|\frac{\mu_{1}-\mu_{2}}{s_{pooled}}\right|$, where ${\text{s}}_{\text{pooled}}=\sqrt{\frac{s_{1}^{2}+s_{2}^{2}}{2}}$.

Figures were designed with Microsoft Excel 2016 and Daniels XL Toolbox 6.60, and most figures represent means +/- standard error of the means, unless otherwise specified.

## Results

### Apnea and righting reflexes

**Fig 3:**
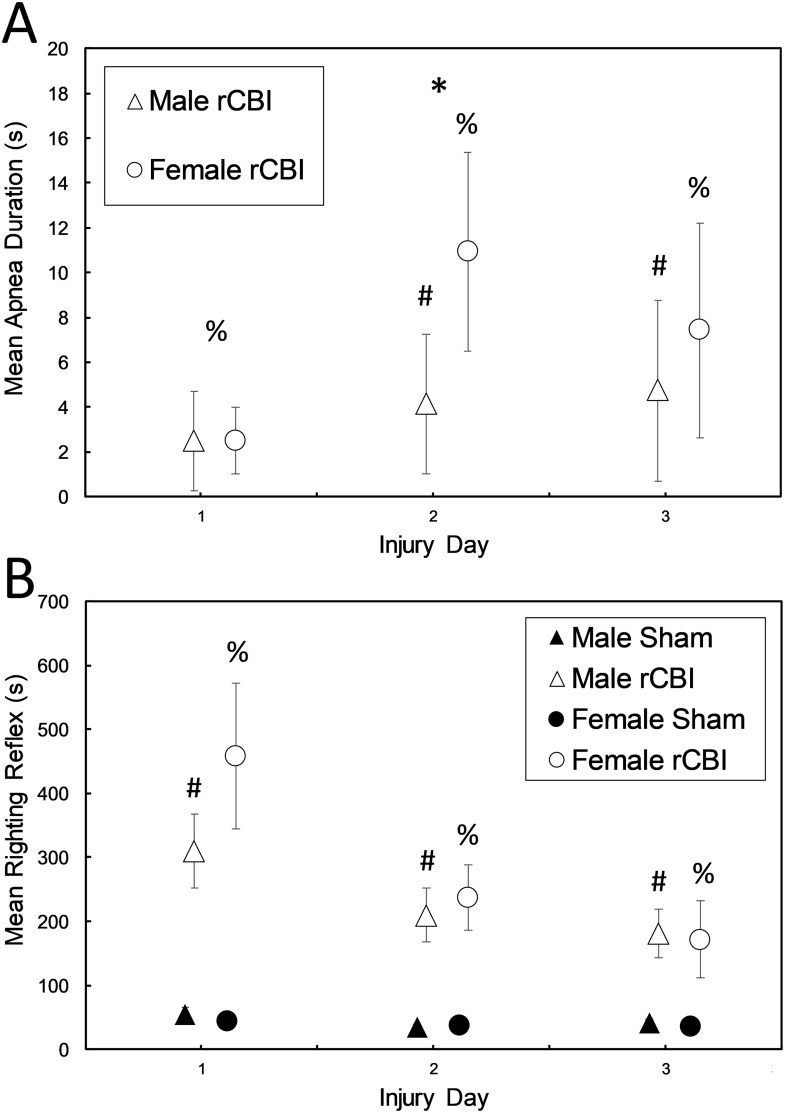
Apnea (A) and righting reflexes (B) following concussive brain injuries. (Shown are means +/- 95% confidence intervals, based on *z*-scores). On Day 1, only the injured female mice had a significant amount of apnea compared to female sham-control mice (**A**, %, Female rCBI > Female Sham, *p* < 0.01). (Note that sham-controls did not experience apnea and all have a value of “0.”) Both sexes had significant durations of apnea compared to sham controls on the second day of injury (%, Female rCBI > Female Sham, *p* < 0.0001; #, Male rCBI > Male Sham, *p* < 0.05); also, female injured mice had longer durations of apnea than male mice (*, Female rCBI > Male rCBI, *p* < 0.05). On the third injury day, both sexes had significant durations of apnea following injury (%, Female rCBI > Female Sham, *p* < 0.0001; #, Male rCBI > Male Sham, *p* < 0.05), but there were no sex differences. Shown in (**B**) are righting reflexes (time to turn from a supine to prone position following cessation of anesthesia) for male and female injured and sham-treated mice. On each of the three injury days, for both males (#, Male rCBI > Male Sham, *p* < 0.0001) and females (%, Female rCBI > Female Sham, *p* < 0.0001), injured mice took significantly longer time to right themselves than sham controls. rCBI, repetitive concussive brain injury.

The incidence of apnea over the three injury days was 20.69%, 31.03% and 27.59% for male mice, and 30%, 60% and 50% for females. Fig 3A shows the duration of apnea following rCBI procedures (no sham-treated mice had apneic episodes). Kruskal-Wallis tests performed for each day found significant differences in the median apnea duration between the four injury and sex groups for each day (Day 1: H(3) = 18.364, *p* < 0.0001; Day 2: H(3) = 42.569, *p* < 0.0001; Day 3: H(3) = 33.745, *p* < 0.0001). On day 1, only the injured female mice had a significant amount of apnea compared to female sham-control mice (*p* = 0.003). On day 2, both male and female injured mice had significant durations of apnea (*p* = 0.046 and *p* < 0.0001, respectively); also, female injured mice had longer durations of apnea than male mice following CBI procedures (*p* = 0.024). On the third injury day, both sexes had significant durations of apnea following injury (*p* = 0.045 and *p* < 0.0001 for males and females, respectively), but the durations of apnea were similar between the sexes (*p* = 0.209).

Righting reflexes following each of the three injuries or sham procedures are shown in Fig 3B. Kruskal-Wallis tests showed that there were significant differences between groups on each day (Day 1: H(3) = 89.670, *p* < 0.0001; Day 2: H(3) = 83.394, *p* < 0.0001; Day 3: H(3) = 78.919, *p* < 0.0001). Follow-up tests confirmed longer righting reflexes in injured mice compared to sham mice of the same sex on each day (Days 1–3: Males and females, *p* < 0.0001), but there were no sex differences on any days in either the sham-treated or injured groups (*p* = 1.0).

### Injury pathology

#### Astrogliosis (GFAP)

**Fig 4:**
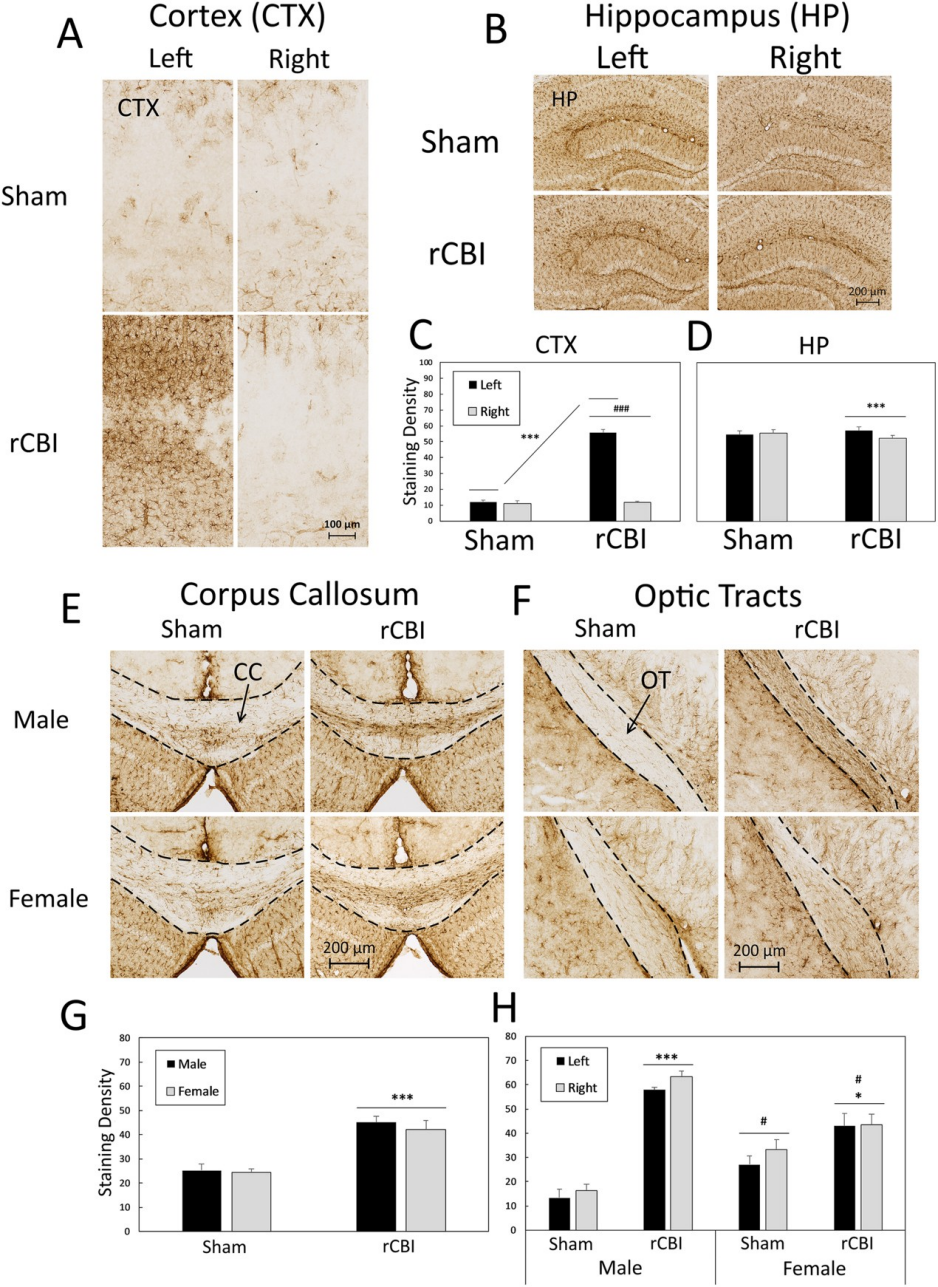
Astrogliosis as observed by GFAP staining following repetitive concussive brain injury (rCBI). All sections shown represent the approximate median value from each group. No sex differences were found in the cortex (CTX; **A & C**) or hippocampus (HP; **B & D** (dentate gyrus, CA1 and CA2/3 regions averaged)) and data are collapsed by sex. GFAP staining was increased on the injured (left) side of the CTX in all mice that sustained rCBI (**A & C**; ***, rCBI > Sham, p < 0.0001). The uninjured side of the brain did not show signs of increased astrogliosis, having significantly reduced staining density compared to the injured side (###, rCBI > Sham, *p* < 0.0001). In the HP, injured mice and sham-treated mice had similar levels of GFAP staining density (**B & D**). In injured mice, however, staining density was higher on the injured (left) side of the brain than on the uninjured side (***, rCBI Left > rCBI Right, *p* < 0.0001). rCBI increased astrogliosis in white matter tracts (**E-H**). The corpus callosum had significantly increased GFAP staining density following rCBI (**E & G**, ***, rCBI > Sham, p < 0.0001). Astrogliosis was also increased in the optic tracts in both male and female mice that sustained rCBI (**F & H**; **F** shows optic tracts from the left (injured) side of the brain), H: ***, Male rCBI > Male Sham (*p* < 0.0001); *, Female rCBI > Female Sham (*p* < 0.05). Sex differences were observed in the optic tracts with males having higher levels of GFAP staining density than females (Male rCBI > Female rCBI; # (*p* < 0.05), Male sham < Female Sham; # (*p* < 0.05). GFAP, glial fibrillary acidic protein; rCBI, repetitive concussive brain injury.

GFAP staining density was significantly increased in the perilesional cortex (CTX) on the injured side of the brain in both male and female mice (Injury x Side interaction: F_1,20_ = 317.41, *p* < 0.0001; Left Sham vs Left (injured) rCBI, adjusted *p* < 0.0001, *d* = 7.19; Left (injured) rCBI vs Right (uninjured) rCBI, adjusted *p* < 0.0001, *d* = 7.63) (Fig 4A & 4C). A four-way ANOVA (injury x sex x region x side) performed on GFAP staining density in the hippocampus (HP) revealed both side by injury (F_1,20_ = 21.68, *p* = 0.0002) and side by region (F_2,40_ = 6.16, *p* = 0.0049) interaction effects. Bonferroni-corrected planned contrasts revealed that staining density was greater on the left side than on the right side in mice that sustained rCBI (*p* < 0.0001, *d* = 0.59) (Fig 4B & 4D). However, there were no differences in staining density between the injured (left) side of the mice that sustained rCBI and either side of the brain of sham-treated mice (*p* ≥ 0.8730). Bonferroni-corrected *t*-tests to follow-up the side by region interaction showed that staining density was equal in the CA1 and CA2/3 regions on both sides (*p* ≥ 0.2276), but GFAP staining density in the DG on both sides was greater than CA1 and CA2/3 staining density on both the left and right sides (*p* < 0.0001, *d* ≥ 1.22; See S1 Table).

Repetitive brain injuries increased astrogliosis in white matter tracts. All mice that sustained rCBI had significantly increased GFAP staining density in the corpus callosum (main effect of injury: F_1,20_ = 49.82, *p* < 0.0001, *d* = 3.00) (Fig 4E & 4G). In the optic tracts, there was an injury x sex interaction effect (F_1,19_ = 21.37, *p* = 0.0002) (Fig 4F & 4H). Both male and female injured mice had greater staining density in the optic tracts than their sex-matched sham controls (*p* < 0.0001, *d* = 6.56 and *p* = 0.0128, *d* = 1.25, respectively). Male injured mice had greater levels of astrogliosis in the optic tracts than female injured mice (*p* = 0.0420, *d* = 1.70), and GFAP staining density was also higher in sham-treated female mice than in sham-treated male mice (*p* = 0.0200, *d* = 1.84).

#### Parvalbumin

**Fig 5:**
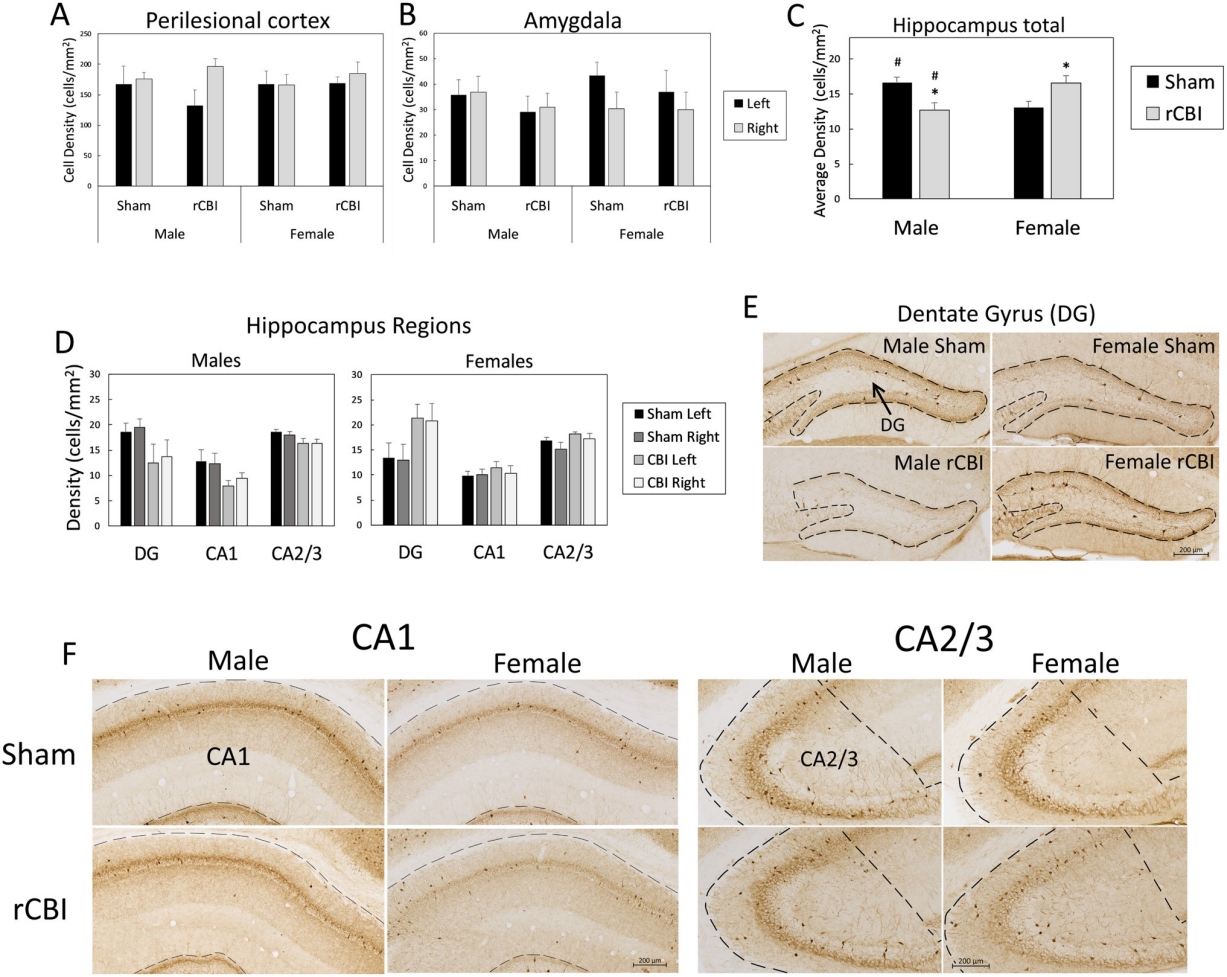
Parvalbumin in the brain following rCBI. All sections shown represent the approximate median value from each group. There were no effects of injury or sex on parvalbumin cell density in the cortex (**A**) or amygdala (**B**). In the hippocampus (**C-F**), rCBI decreased the overall parvalbumin cell density in males (*, Male Sham > Male rCBI, *p* < 0.05), but cell density was increased in females that sustained injuries (**C**; *, Female rCBI > Female Sham, *p* < 0.05). Female injured mice also had a greater parvalbumin cell density than male injured mice (#, Female rCBI > Male rCBI, *p* < 0.05), and there were also sex differences between sham-treated animals, with male mice having a greater density of parvalbumin-reactive cells than females (#, Male Sham > Female Sham, *p* < 0.05). Although there was no statistically significant interaction effect with region, the sex differences were most apparent in the DG. rCBI, repetitive concussive brain injury; DG, dentate gyrus.

Parvalbumin cell density in the perilesional CTX and the amygdala was not affected by injury, sex or side of brain (F_1,40_ ≤ 2.46, *p* ≥ 0.1245) (Fig 5A & 5B). There were also no effects of any factors on parvalbumin cell density in the auditory CTX (F_1,38_ ≤ 3.87, *p* ≥ 0.0565; See S1 Table). In the hippocampus, there was an injury by sex interaction effect on the density of parvalbumin cells (F_1,20_ = 10.94, *p* = 0.0035) (Fig 5C). Bonferroni-corrected planned contrasts revealed the following group differences: Male rCBI < Male Sham (*p* = 0.0141, *d* = 0.73); Female rCBI > Female Sham (*p* = 0.0450, *d* = 0.60); Male rCBI < Female rCBI (*p* = 0.0180, *d* = 0.62); Male Sham > Female Sham (*p* = 0.0284, *d* = 0.72). Although the injury by sex by region interaction effect was insignificant (F_2,19_ = 2.63, *p* = 0.0983), the injury effect was most apparent in the dentate gyrus (Fig 5D & 5E).

### Open field (OF) test

#### Total distance traveled

**Fig 6:**
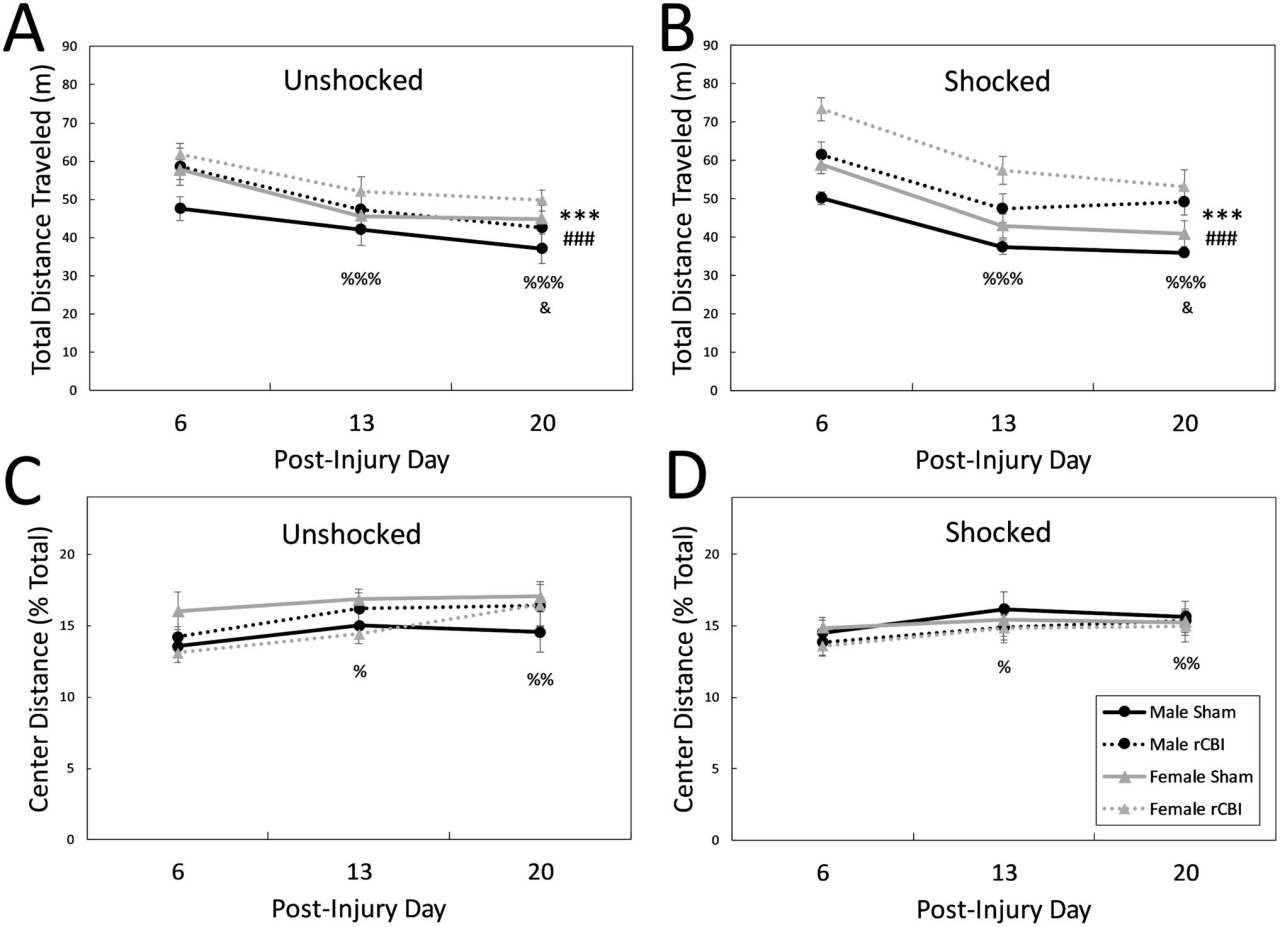
Total distance traveled (A & B) and activity in the center zone (C & D) in the OF arena for unshocked and shocked mice. There were main effects of injury (**###**, rCBI > Sham, *p* < 0.0001) and sex (***, Female > Male, *p* < 0.0001) on the total distance traveled in the OF (**A & B**). There was also an effect of day; all mice were less active on days 13 and 20 when compared to the first exposure to the test on day 6 (%%%, Day 6 > Days 13 and 20, *p* < 0.0001), and there was a decrease in activity between days 13 and 20 (**&**, Day 13 > Day 20, *p* < 0.05). The distance traveled in the center of the arena (**C & D**) was also affected by day; mice increased their exploration of the center zone on days 13 (**%**, Day 13 > Day 6, *p* < 0.05) and 20 (**%**, Day 20 > Day 6, *p* < 0.01) compared to day 6. There were no effects of injury, sex, or shock on center activity. OF, open field; rCBI, repetitive concussive brain injury.

Analysis of total activity in the OF arena on days 6, 13 and 20 following injury revealed no main effect of shock (F_1,111_ = 0.42, *p* = 0.5173) or interaction effects between the four factors (p ≥ 0.0805), but there were main effects of injury (F_1,111_ = 21.50, *p* < 0.0001), sex (F_1,111_ = 12.83, *p* = 0.0005), and day (F_2,222_ = 106.00, *p* < 0.0001). Overall, females ambulated more in the OF than males (*d* = 0.47), and injured mice were hyperactive compared to sham controls (*d* = 0.70) (Fig 6A). In addition, all mice were less active in the OF on days 13 and 20 compared to their first exposure to the arena on day 6 (*p* < 0.0001, *d* = 0.91 and 1.08, respectively); there was also a significant, but small, decrease in distance traveled between days 13 and 20 (*p* = 0.0475, *d* = 0.16).

#### Center activity

There were no effects of sex (F_1,111_ = 0.16, *p* = 0.6932), injury (F_1,111_ = 1.02, *p* = 0.3144), or shock (F_1,111_ = 0.55, *p* = 0.4586) on the distance traveled in the center of the OF arena (expressed as a percentage of the total distance traveled), nor were there any interaction effects between sex, injury, shock, and/or day (F ≤ 2.24, *p* ≥ 0.1377). There was a main effect of day on center activity (F_2,222_ = 6.85, *p* = 0.0013); distance traveled in the center increased on days 13 and 20 compared to the first exposure on day 6 in all mice (*p* ≤ 0.0124, *d* ≥ 0.33, Fig 6B).

### Fear Conditioning (FC)

#### Training

**Fig 7:**
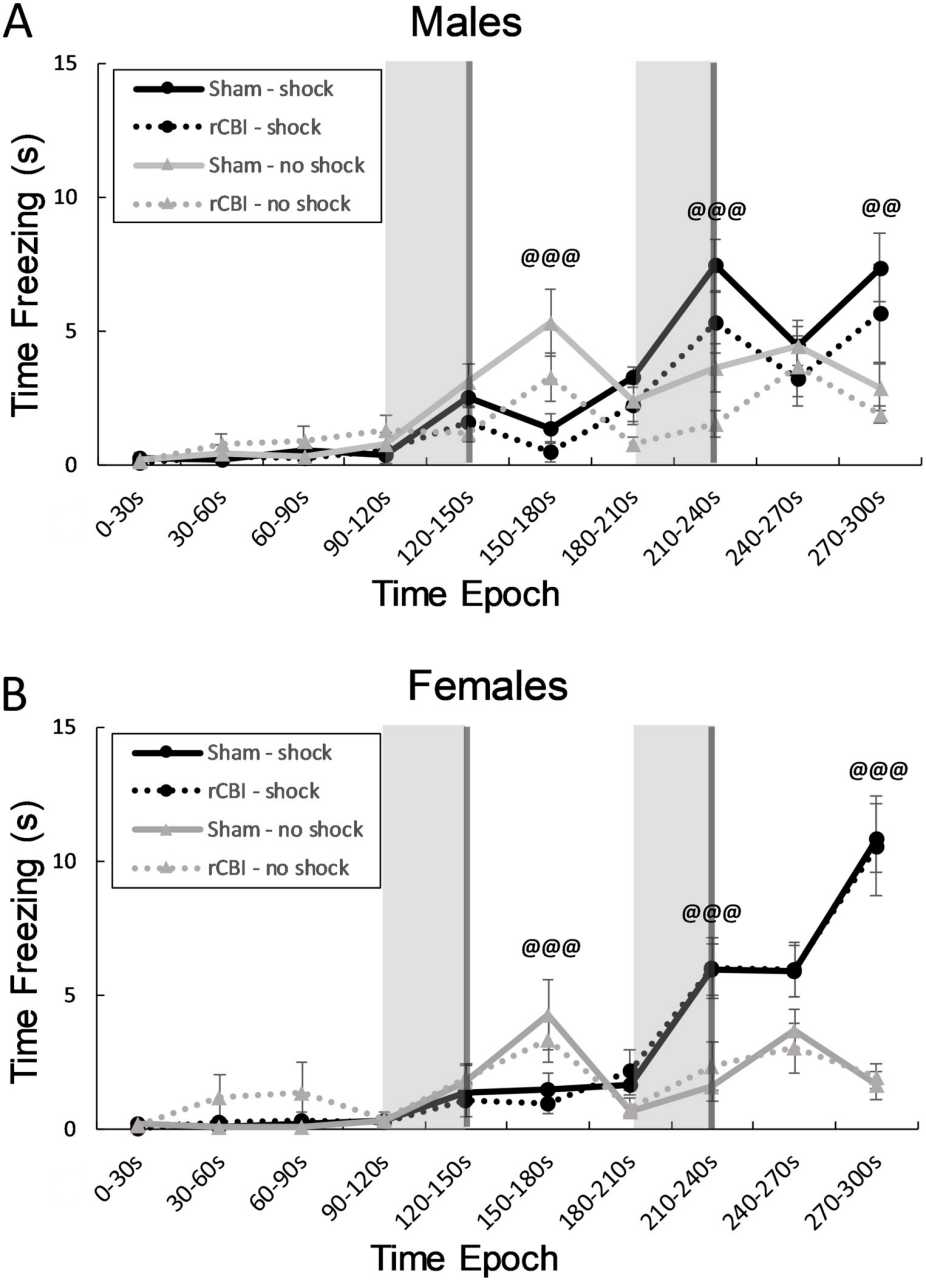
Freezing behavior during pairing of a neutral stimulus (tone) with an aversive stimulus (foot shock). Regions with light gray shading indicate times during which tone was presented; tones co-terminated with a 2-second foot shock (darker gray bar). During the 30-second time period following the first tone (150-180s), shocked mice froze less than unshocked controls (@@@; **A** & **B**, Unshocked > Shocked, *p* < 0.0001). However, during the second tone (210-240s) and during the last time period (270-300s), shocked mice displayed more freezing behavior than mice that did not receive foot shocked paired with the tones (@@@, Shocked > Unshocked, *p* < 0.0001, @@, Shocked > Unshocked, *p* < 0.01). rCBI, repetitive concussive brain injury.

There were no effects of injury, or interaction effects of injury with any other factors (F ≤ 1.86, *p* ≥ 0.0551), on freezing behavior during the tone/shock association stage of FC one week following CBI. Due to a significant sex x time x shock interaction effect on the amount of freezing during this trial (F_9,957_ = 2.28, *p* = 0.0155), separate three-way (injury x shock x time) ANOVAs were performed for males (Fig 7A) and females (Fig 7B). Significant time by shock interaction effects were found in both male (F_9,473_ = 11.19, *p* < 0.0001) and female (F_9,482_ = 15.86, *p* < 0.0001) mice. Male and female unshocked mice displayed more freezing behavior (shocked mice were more active) during the 30 seconds immediately following the first tone/shock pairing (150-180s; *p* < 0.0001, *d* = 1.10 and 1.20 for males and females, respectively), but shocked mice froze more during the second tone (210-240s; p < 0.0001, *d* = 1.14 and 1.24 for males and females, respectively) and during the last 30 seconds of the test (270-300s; males, *p* = 0.006, *d* = 0.96; females, *p* < 0.0001, *d* = 1.68).

#### Context test

**Fig 8:**
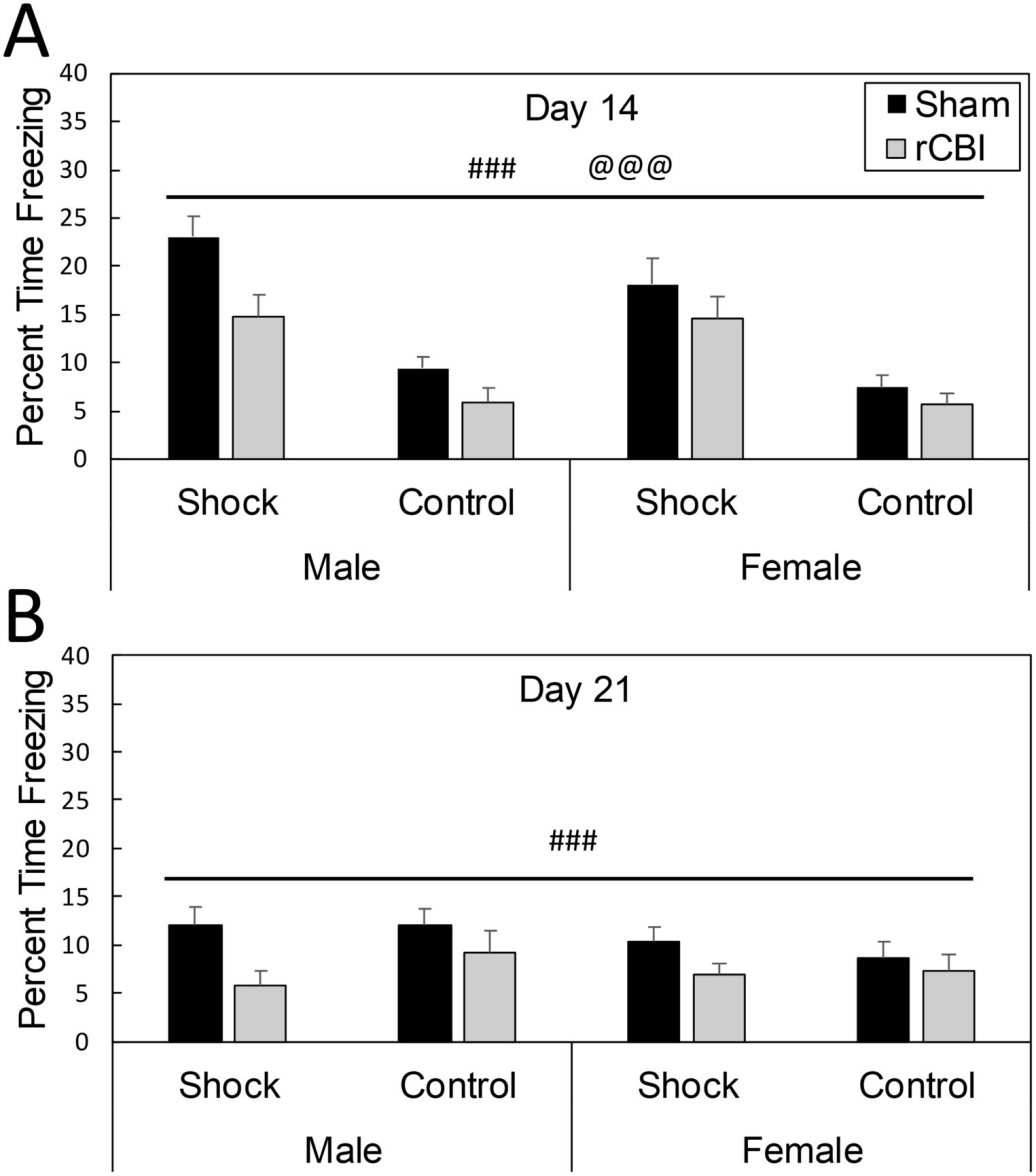
There was a main effect of injury (###, rCBI < Sham, *p* < 0.0001) on time freezing in the FC context test (A & B), performed 14 and 21 days following rCBI (7 and 14 days following FC tone/shock association). Injured mice froze less time when placed back into the context in which they had previously received foot shocks. Shock only had an effect on freezing behavior 7 days following tone/shock association (14 days following rCBI; **A**); mice that had been shocked in the visual context froze a greater duration of time on that day than control mice that had not been shocked (@@@, Shocked > No Shock). FC, fear conditioning; rCBI, repetitive concussive brain injury.

The context test was performed 7 and 14 days following training/association (14 and 21 days following rCBI or sham treatments). A significant main effect of injury was found (F_1,110_ = 16.82, *p* < 0.0001, *d* = 0.60), with mice that had sustained rCBI freezing less than sham controls in response to the context in which they had previously received the tone alone, or shock tone/shock pairings (Fig 8A & 8B). There was also a shock by day interaction effect (F_1,108_ = 60.03, *p* < 0.0001); shocked mice froze more than unshocked controls during the first exposure to the context (*p* < 0.0001, *d* = 1.39, Fig 8A), but these groups displayed equivalent levels of freezing one week later (*p* = 1.0, Fig 8B).

#### Cue test

**Fig 9:**
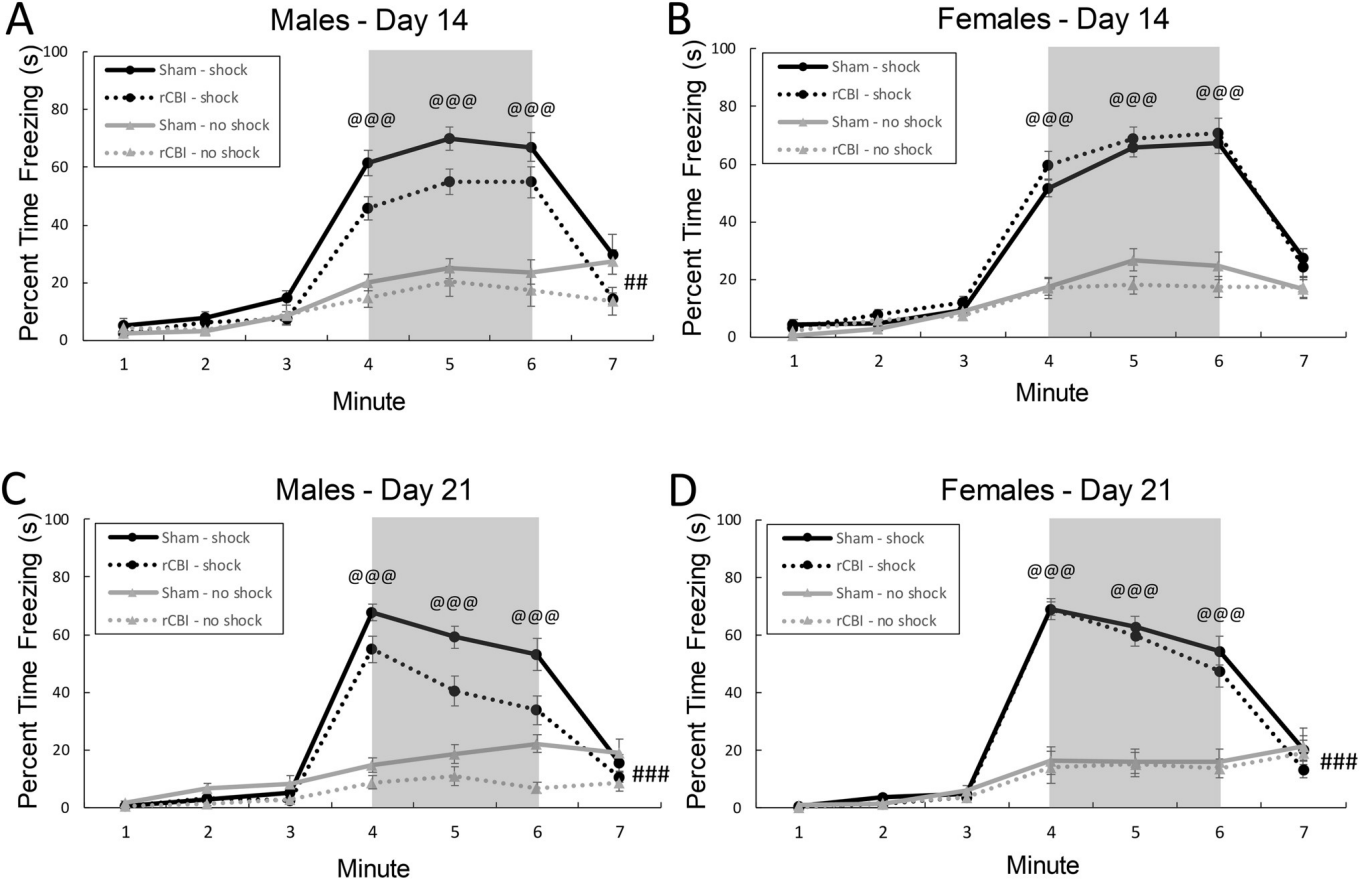
Freezing behavior during a cue test (with the tone presented during minutes 4–6, regions shaded in gray) on days 14 (A & B) and 21 (C & D) following rCBI (days 7 and 14 following tone/shock association. Shock status had a significant effect on amount of freezing behavior during the minutes in which the tone was presented (4–6; @@@, Shock > No Shock, *p* < 0.0001), on both days 14 and 20 following rCBI (A-D). On day 14, there was an effect of rCBI in male mice (**A**), with injured mice displaying less freezing behavior than sham controls during the test session (##, Male rCBI < Male Sham, *p* < 0.01). On day 21, there was a main effect of rCBI, with all injured mice freezing less during the test session than sham controls (###, Sham > rCBI, *p* = 0.0001). rCBI, repetitive concussive brain injury.

Significant minute by shock (F_6,618_ = 53.72, *p* < 0.0001) and injury by sex (F_1,141_ = 4.43, *p* = 0.0371) interaction effects were found for freezing behavior during the cue test on day 7 following tone/shock association (day 14 following rCBI or sham procedures) (Fig 9A & 9B). Sham-treated male mice froze more during the test than injured mice did (*p* = 0.0076, *d* = 0.27; Fig 9A), but there was no effect of injury in female mice (*p* = 1.0; Fig 9B). Shocked and unshocked mice displayed equivalent freezing behavior during the baseline of the test (minutes 1–3; *p* ≥ 0.3356) and during the final minute of the test following the tone (minute 7, *p* = 0.478), but shocked mice froze more during all three minutes of the tone presentation (minutes 4–6, *p* < 0.0001, *d* > 2.39).

On day 14 following association (day 21 following injury or sham procedures) (Fig 9C & 9D), the injury by sex interaction effect neared significance (F_1,150_ = 3.59, *p* = 0.0599), with the injury effect continuing to be more pronounced in males (Fig 9C). A main effect of injury was significant (F_1.150_ = 15.55, *p* = 0.0001, *d* = 0.23), with mice that had sustained rCBI freezing less during the test than sham controls (Fig 9C & 9D). There was also a minute by shock interaction effect (F_6.649_ = 98.69, *p* < 0.0001); previous shocks had no effect on freezing behavior during minutes 1–3 (baseline) of the test (*p* = 1.0) or the final minute (*p* = 1.0), but mice that had previously associated the shock with the tone froze significantly more during the tone (minutes 4–6) than mice that had not been shocked previously (*p* < 0.0001, *d* ≥ 1.73) (Fig 9C & 9D).

### Elevated zero maze (EZM)

**Fig 10:**
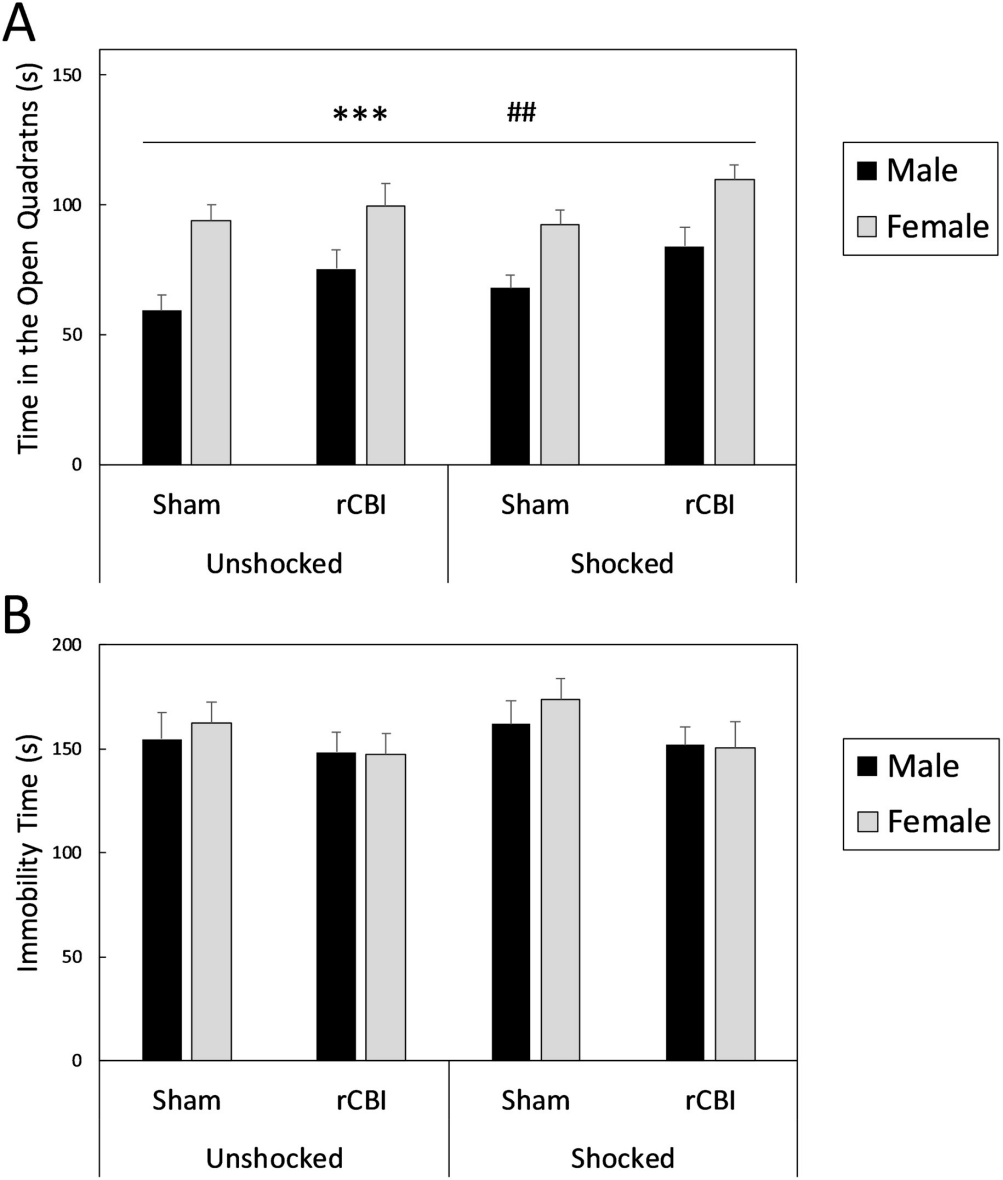
Anxiety- and depressive-like behaviors in the EZM (A) and TST (B), respectively. There were significant effects of both injury (##, rCBI > Sham, *p* < 0.01) and sex (***, Female > Male, *p* < 0.0001) on the time spent in the open (anxiogenic) regions of the EZM, with mice that had sustained rCBI and female mice spending more time in the open quadrants of the apparatus than sham-treated and male mice, respectively (**A**). In the TST (**B**), there were no effects of injury or sex on the amount of time spent immobile. Shock status did not influence behavior in either test. EZM, elevated zero maze; TST, tail suspension test; rCBI, repetitive concussive brain injury.

In the EZM, there were significant main effects of injury (F_1,118_ = 8.89, *p* = 0.0035, *d* = 0.52) and sex (F_1,118_ = 34.03, *p* < 0.0001, *d* = 1.05) on the amount of time spent in the open quadrants of the maze (Fig 10A). Injured mice and female mice explored the open (anxiogenic) quadrants more than sham-treated and male mice, respectively. There were no significant effects of shock (F_1,111_ = 1.92, *p* = 0.1680) or interaction effects between the three factors (F_1,111_ ≤ 0.37, *p* ≥ 0.5465).

### Tail suspension test (TST)

There were no effects of injury, sex, shock, or interactions among the three factors (F_1,118_ ≤ 2.99, *p* ≥ 0.0867) on immobility in the TST (Fig 10B).

## Discussion

### Summary of behavioral and neuropathological findings following rCBI

Table 2 summarizes the principal effects from rCBI. Assessed by measurement of the freezing response in the FC paradigm, parietal rCBI impaired contextual fear memories (Fig 8). However, the effect of injury on freezing behavior in response to the auditory cue was sex-dependent. In comparison to sham-controls, injured males, but not females, exhibited a decrease in freezing behavior during presentation of the cue (Fig 9). The injury was further characterized behaviorally by hyperactivity in the OF test (Fig 6A), and a greater level of exploration in anxiogenic (bright and open) regions of the EZM (Fig 10A); these effects of injury were independent of sex. However, there were main effects of sex on some behavioral parameters: females were more active in the OF arena and were more likely to explore the open and brightened quadrants of the EZM.

**Table 2:** Summary of the effects of rCBI on behavior and neuropathology.

Sex	Freezing Response		Behavioral Changes in Injured Mice	Neuropathology	
	Contextual FC Hippocampus/amygdala	Cued FC Amygdala		GFAP	Parvalbumin
Males	Impaired (*p* < 0.0001)	Day 7: Impaired (*p* = 0.0076, *d* = 0.27) Day 14: Impaired (*p* = 0.0001)	OF hyperactivity (*p* < 0.0001) EZM ↓ anxiety (*p* = 0.0035) No change for TST	Astrogliosis in CTX (p < 0.0001), CC (p < 0.0001), OT (male: *p* < 0.0001, *d* = 6.56; female: *p* = 0.0128, *d* = 1.25), and HP on injured side (*p* < 0.0001, *d* = 0.59)	Decreased in hippocampus (*p* = 0.0141, *d* = 0.73) No change in cortex or amygdala
Females	Impaired (*p* < 0.0001)	Day 7: No change Day 14: Impaired (*p* = 0.0001)	OF hyperactivity (*p* < 0.0001) EZM ↓ anxiety (*p* = 0.0035) No change for TST		Increased in hippocampus (*p* = 0.0450, *d* = 0.60) No change in cortex or amygdala

Cohen’s *d*-values are reported for males and females only where significant injury x sex interaction effects were found. For main effects of injury, only *p*-values are reported; Cohen’s *d*-values are found in the main text. rCBI, repetitive concussive brain injury; FC, fear conditioning, OF, open field; EZM, elevated zero maze; TST, tail suspension test; GFAP, glial fibrillary acidic protein; CTX; cortex; CC; corpus callosum; OT, optic tracts; HP, hippocampus

Pathologically, the injury model was characterized by increased astrogliosis (GFAP staining; Fig 4) in the cortex at the site of the injury, as well as in white matter tracts including the corpus callosum and optic tracts as previously described [7, 10]. Interestingly, measured staining in the sham-treated mice differed: female mice exhibited greater stain density than males. Conversely, following rCBI, the male mice exhibited higher levels than females. The source of these differences are unknown. There appear to be no reports suggesting a sex difference in GFAP staining in the optic tract. The number of parvalbumin-expressing interneurons was unaffected by injury in the cortex and amygdala, but the effect of injury on the density of the labeled interneurons in the hippocampus was sexually dimorphic, with injury reducing the number of interneurons in males, but increasing the number of parvalbumin-expressing cells in females (Fig 5). Under control conditions it appears female C57Bl/6J mice have slightly more parvalbumin neurons [37], but levels are reportedly similar in both sexes in the hilus of the hippocampus [38]. The increase in paravalbumin levels in females following rCBI is unknown, but exercise is known to increase paravalbumin neuron number in the hippocampus and female mice in general, and as observed here after TBI, are more active [39].

### rCBI impaired contextual fear memories

The neural substrate of FC has received extensive study [17], with the hippocampus and amygdala ascribed as two primary brain regions implicated in contextual FC test performance [40]. Contextual fear conditioning is often employed as a specific behavioral test of hippocampal function as this region is considered a critical component for fear-related learning [11, 14, 16]. Deficits in contextual fear memory following experimental brain injury have been described previously in male mice following parietal rCBI [11], and in male mice following diffuse TBI from lateral fluid percussion (LFP) injury targeted to the parietal cortex and underlying dorsal hippocampus [14, 16, 41, 42].

Using an established model of parietal region rCBI [7, 10, 11], this study found there was an injury-dependent decrease in freezing to the context following shock/context association. The differences are not likely due to a deficit in the production of the freezing response, as sham and injured mice had equal freezing responses during the training/acquisition trial. Gross hippocampal damage (obvious tissue loss) was not observed, although there was a very small increase in astrogliosis in the hippocampus on the injured side of the brain compared to the uninjured side. TBI-induced deficits on other hippocampal-dependent tasks such as the Morris water maze have also been reported in the absence of extensive hippocampal pathology, and it has been suggested that subtler microstructural changes following injury could be responsible for functional impairments [43].

Parvalbumin-immunoreactive (PV-IR) interneurons comprise a large proportion of inhibitory GABAergic interneurons in the hippocampus (and amygdala), and are intricately involved in the neuronal circuitry required for FC acquisition, storage and retrieval [44–47]. Loss of PV-IR cells in the dentate gyrus, as observed here, has been reported previously in male rodents following LFP [48, 49] or concussive brain injury [50]. The loss of these inhibitory interneurons has been associated with increased dentate gyrus excitability [48] and a chronic, progressive loss of synaptic inhibition [49]. Controlled cortical impact (CCI) resulted in an overall loss of GABAergic interneurons (as assessed by GAD-67 staining) in the hippocampus of rats, together with reduced GABAergic synaptic transmission and deficits on a passive avoidance task [13]. Both neuronal loss and neuronal dysfunction (e.g., impaired long-term potentiation, changes in cell excitability, reductions in axon conduction velocities) have been reported in the hippocampus in many rodent TBI models [11, 14, 51–58]. Witgen and colleagues demonstrated contextual fear deficits in rats that had sustained LFP injury; these deficits were associated with both profound neuronal loss throughout the hippocampus as well as changes in synaptic efficacy and evoked currents [14].

There were no injury-induced differences in the perilesional or auditory cortex or, as noted, the amygdala in the density of parvalbumin-immunoreactive (PV-IR) interneurons, but there was a sex-dependent loss of PV-IR interneurons in the hippocampus: PV-IR cells were reduced in injured male mice, but female injured mice had more PV-IR cells in the hippocampus than sham-controls. The sex difference in PV-IR cell changes in the hippocampus we observed is difficult to explain in the context of the current data set. Both male and female injured mice were impaired in contextual fear conditioning; sex differences were observed in the cue test which is largely independent of hippocampal-function. Female advantages on hippocampal-dependent functional tests (Barnes maze, Morris water maze) following experimental TBI have been reported [7, 59], and it is possible that the sexually dimorphic changes in hippocampal PV-IR cell populations and resulting changes in hippocampal function reported by other investigators could explain differences in post-TBI performance on those other tasks. Electrophysiological studies of hippocampal function following TBI including both sexes would help answer these questions.

Importantly, contextual FC deficits must be considered a consequence of a brain injury more diffuse than potential damage to a single region (hippocampus); there are many brain regions implicated in contextual FC performance secondary to the hippocampus [60]. Lesion studies have demonstrated that the ability to form new contextual memories is spared when the hippocampus is damaged prior to association trials [61, 62], an experimental design employed in most (including the current) translational TBI studies. This suggests that TBI-induced deficits in context memory may result from damage to brain regions other than the hippocampus. A full discussion on the relative role of the hippocampus in anterograde contextual memory goes beyond the scope of this paper, but it is believed that during CS/US association an intact hippocampus allows for a more rapid assimilation of multiple elements of the testing environment into a “gestalt” representation of the context [63]. With hippocampal impairment, it is suggested that the neocortex, which normally works together with the hippocampus, is able to form the contextual representation albeit much more slowly [64].

Finally, the perilesional/parietal cortex damage, evident by astrogliosis, could have led to deficits in contextual encoding during the association trial. As discussed by Hogg and colleagues [65], damage to the parietal cortex can cause many functional deficits (e.g., spatial localization deficits [66], spatial neglect [67]) that may lead to a failure to properly encode the features of the environment. In addition, the parietal cortex contains neurons that respond to a single modality or are multimodal, responding to any single modality (e.g., visual, tactile), or combination of, sensory modalities [68]. Although the mechanisms of multimodal integration are currently unclear, neurons able to assimilate information about multiple features in the environment are uniquely suited for participation in contextual encoding during FC.

### rCBI impaired cued fear memory that was sex dependent

While we found no changes in PV-IR cell density in the amygdala, male, but not female mice, had a deficit in the cue test following TBI. Decreases in response to an auditory CS have been reported following experimental TBI [15, 69, 70], although it should be noted that many other investigators have found no differences in the cue test following TBI (e.g., [41, 71–74]). The freezing response to the cue (as a conditioned stimulus) in the fear conditioning paradigm is directly associated with activity in the amygdala [40, 75]. Palmer and colleagues reported impaired freezing responses in response to a cue in male mice following LFP; this altered behavior was associated with amygdala circuit dysfunction (decreased network excitability), and suggested to be the result of injury-induced disturbance in the balance between inhibition and excitation [15]. Likewise, an overall loss of GABAergic interneurons and reduced inhibitory synaptic transmission has been described in the amygdala of male rats following mild CCI [12].

### rCBI induces hyperactivity in the open field

In the OF, a test that assesses exploration and general motor abilities, mice that sustained rCBI ambulated greater distances overall than sham-treated animals. Hyperactivity has been previously reported in translational TBI studies employing severe contusive injury models such as controlled cortical impact (CCI) (e.g., [25, 76, 77]), more severe closed-head methods [78, 79], or repetitive mild closed-head injuries [10, 80, 81]. These injuries may result in either gross lesions or more subtle damage to the hippocampus, and discrete hippocampal lesions result in OF hyperactivity [82–84], However, this is only one of many experimental manipulations resulting in an increase in general locomotor activity [85], and as most experimental models of TBI induce diffuse as well as focal injuries, changes in arousal and activity could be the outcome of insult to many regions, including parietal cortex.

### Disinhibition in the elevated zero maze following rCBI

Mice with rCBI spent a greater amount of time in the bright and exposed zones of the EZM, suggesting reduced anxiety in these animals compared to controls. Some translational TBI studies employing mild and/or repeated concussive models in mice report the same results in the EZM or the elevated plus maze (EPM; a very similar test) [10, 80, 86–88], whereas others have found increased anxiety-like behaviors in these tests following TBI [89–93] or no differences between groups [72, 94–97]. Decreases in anxiety-like behavior in the EPM or EZM are sometimes described as “behavioral disinhibition” (e.g., [87, 88, 98]). This phenotype can be induced with hippocampal lesions (e.g., [99, 100], and is often described in rodents following more severe injuries that result in overt damage to the hippocampus, such as CCI [24, 101, 102]. More recently, a longitudinal study following CCI in mice showed injury-induced anxiety in the EZM at a more acute time point (1 wk) and decreased anxiety later (5 wks), with OF hyperactivity at both time points [103]. GABAergic inhibition was upregulated in the basolateral amygdala 9 wks following injury; Almeida-Suhett and colleagues reported a decrease in GABA immunoreactivity in the basolateral amygdala that corresponded with increased anxiety-like behaviors in rats [12]. Further studies are required to determine the potential time course and neural mechanisms underlying TBI-induced anxiety-like behaviors.

### rCBI does not affect depressive-like behavior in the tail-suspension test

In this rCBI model, we found no effects of injury on depressive-like symptoms as measured by the TST, consistent with other studies employing the TST or forced-swim test (FST) to measure behavioral despair after similar injuries [72, 93, 94, 104]. Although other investigators have reported increased symptoms of behavioral despair [8, 73, 105, 106] or anhedonia as measured by the sucrose preference test after rCBI ([73], (but see [107]), it had been noted that like anxiety, modeling post-TBI depression in animal models has been challenging and results have been inconsistent [24, 108]. TBI studies including assessments of behavioral despair (e.g., TST and FST) and anhedonia (sucrose preference test (SPT)) are relatively few compared to those measuring motor and cognitive deficits, and are widely disparate in terms of experimental details such as TBI model and time point after injury at which behavioral testing is performed [108]. As depression is one of the most common clinical complaints following mild TBI [109, 110] and sports-related repetitive brain injuries (i.e., chronic traumatic encephalopathy) [1, 111–113], greater efforts should be made in translational studies to address appropriate modeling and treatment of these symptoms.

### Female mice are more active and willing to explore anxiogenic locations

Independent of injury status, female mice in this study traveled greater distances in the OF and explored the open/brighter zones of the EZM more than male mice. These results are consistent with previous data from our laboratory [24, 25, 33]. It has been long noted that female mice overall demonstrate greater activity levels than males in an OF environment [114, 115], but ultimately results are inconsistent and likely dependent on specific laboratory conditions such as lighting level [116] or more subtle aspects of the environment [117]. Many reviews have also noted that contrary to the human condition in which females are more likely to suffer from anxiety disorders [118], female rodents often display fewer anxiety-like behaviors in pre-clinical anxiety assessments that rely on inherent approach/avoidance conflict [115, 119, 120]. However, caution should be taken with interpretation as activity levels could be a confounding variable [121–124].

### Morphological considerations

With the exceptions where performance was better in females, the preponderance of evidence suggests there were no extraordinary sex differences in functional outcomes (Table 2). Differences noted in the present study could have arisen from using the same impact parameters in the rCBI with no adjustments for differences in body size of male and female mice; a significant concern of “scaling” when researchers attempt to make species comparisons or consider the compatibility of injury levels across species in TBI modeling [125]. Likewise, there is a possibility that the injury “level” was not equivalent due to sex differences in functional responses after impacts (changes in blood flow, inflammation, metabolic responses, hormone status) as well as structural differences. The duration of apnea was greater in the injured females than in injured males and thus size and the aforementioned factors may be important. Related to structural differences, for example, skull anatomy could be a factor. Kawakami and Yamamura measured a variety of skull characteristics in 12 week old male and female mice [126]. Analyses of data in their Table 1 indicated no differences between male and female C57Bl/6J mice for parietal bone length or frontal bone width. However, analysis indicated the sexes differed for interparietal bone width (Males, n = 6, mean width 8.217 ± 0.172 mm (mean ± standard deviation) vs Females 7.867 ± 0.115 mm). Likewise, Čsanády, and Mošanský evaluated a number of parameters in *Mus musculus* adult skulls, and reported no sexual size dimorphism [127]. Kupina and colleagues evaluated skull thickness in three CF-1 male (6–8 weeks old) and three female (11–12 weeks old) mice of approximate equivalent weight and reported no differences [128]. Maga and colleagues recently examined variations in skull size. This group compared the skull shape of male and female A/J × C57BL/6J crossed mice and reported the greatest differences were cranial vault curvature and in the length of the basicranium [129], mean skull size was smaller in females (Maga, personal communication), and facial width may be smaller [130]. However, these differences were considered trivial for the laboratory mouse compared to dimorphic sex differences in primates [129]. A second potential confound relates to the overall body size differences of approximately 40%. However, while the male and female mice differed significantly in body weight, the relative differences between male and female brain volume in adult rodents is reportedly on the order of 2.5% [131], although individual regions of the adult mouse differ by sex [132], and this could have a different effect on the biomechanical properties of injury. Nonetheless, previous data indicated no significant difference in cortical thickness changes (although females in general had a slightly thinner cortical layer) [7]. This was confirmed in a publication just appearing [133]. Also, no differences across sex for lesion volume were reported after a more severe injury (CCI) in two separate publications [24, 25]. Structural as well as physiological differences, including endocrine and immune factors, underscore this is a complex question [134].

### Conclusions

In summary, we have reported deficits in contextual fear conditioning following rCBI in male and female mice, which may be due to functional hippocampal changes or more diffuse and global, including parietal cortex, damage. TBI-induced reductions in freezing in response to the auditory cue (amygdala-dependent) only occurred in male mice; these changes are consistent with functional changes in the amygdala following TBI reported by other investigators. Further electrophysiological studies are called for to investigate mechanisms of sex differences in amygdala- and hippocampus-dependent behaviors, as well as the sex difference we report in PV-IR cell density in the hippocampus following rCBI.

Despite sex differences reported in the development of neuropsychiatric symptoms following TBI in clinical populations, the EZM and TST failed to find TBI-related differences between male and female mice in symptoms of anxiety and depression, respectively. Pre-clinical TBI studies in mice with functional assays for anxiety and depression including both sexes are limited. A recent report showed that blast-induced TBI resulted in anxiety (assessed in the EPM) in both male and female mice [29], but CCI was previously shown to cause fewer anxiety-like symptoms in females [24]. No sex differences were reported in the FST or the SPT following CCI [24] or in the TST following rCBI [10]. The inconsistencies in results and complexities of the literature describing sex differences in stress responses have been discussed [115, 135], and it has been noted that many studies find that male rodents may be more prone to depression-like states, and rodents may not be adequate models for translational studies of mood disorders. More specifically, more studies that are inclusive of both sexes with functional assessments of neuropsychiatric symptoms are needed.

The brain regions and circuits directly responsible for behaviors such as OF, EZM and TST are less defined than those for FC, but the tests, particularly EZM and TST, assess symptoms of high clinical prevalence (anxiety and depression, respectively) observed following acquired TBI. Thus, pre-clinical studies of emotional behaviors that include both sexes together with physiology to understand changes in the brain regions and functional circuits underlying those behaviors, are critical for our understanding of the development of neuropsychiatric symptoms following TBI.

## Supporting information

S1 Table Results of pathology and behavior. Means and standard deviations for all reported parameters.
(XLSX)
